# Narrating the self-injured body

**DOI:** 10.1136/medhum-2013-010488

**Published:** 2014-05-08

**Authors:** Amy Chandler

**Keywords:** Mental health care

## Abstract

Illness narratives have traditionally been used as a conceptual tool for exploring experiences of chronic illness or disease. In this paper, I suggest that Frank's typology of illness narratives (chaos, restitution and quest) also offers an illuminating approach to analysing accounts of self-injury, demonstrating the diverse ways in which self-injury is practiced, experienced and narrated. Drawing on 24 narrative interviews with 12 people who had self-injured, I focus on participants’ accounts of their self-injured bodies. The approach is phenomenological, and concerned with talk about the experience of living with and in a body that has been marked by self-injury. Thus, the *act* of self-injury is not the sole focus, and particular attention is paid to accounts of the bodily aftermath: scars, marks and wounds. Scars left by self-injury can be seen as communicative, and the analysis developed here demonstrates some of the various ways that these marks may be read. Attending to these diverse narratives can contribute to the provision of compassionate, non-judgemental care for patients who have self-injured. Further, highlighting the existence of different ways of narrating the self-injured body may offer an optimistic resource for people who have self-injured.

## Introduction

People who self-injure can be seen to occupy an uncertain position, one which unsettles notions of sanity and madness, and dramatically breaches imagined boundaries between physical and mental health. Self-injury^1^ is a contested practice, with long-standing debates regarding how it should be named and categorised.^1^ ^2^ In part, this complexity arises from the diverse meanings that are attached to self-injury, as identified by a growing body of qualitative research with people who have self-injured and clinical practitioners who suggest self-injury is variously a method of managing emotions; self-punishment; interpersonal manipulation; coping mechanism; attention seeking; emotional expression; or communication of distress.^3–7^ Evidently, there are tensions among these meanings, and this may particularly arise when contrasting the views of healthcare staff with patients.^8–10^ The existence of such tensions underlines the importance of attending to the diverse narratives of individuals who self-injure in order to support compassionate, non-judgemental clinical responses.^11^ ^12^

Illness narratives have become a widely used approach in scholarly work seeking to illuminate the importance of patient experience.^13–17^ The concept and use of illness narratives have been much debated within and without the medical humanities.^18–21^ In particular, concerns have been raised about the power of illness narratives to supply access to patient's ‘inner worlds’, while others have questioned the framing of narrative as a ‘universal’ mode of communication and experience.^19^ ^21^ Such critiques are not to be dismissed and underline the importance of approaching narrative analysis with care. A great strength of narrative analysis is that it enables examination of the complex ties between individual stories and wider cultural contexts.^22^ ^23^ This need not involve any attempt to access the ‘inner world’ of people's experiences, and this is certainly not the aim of this paper.^24^ Rather, in what follows, I focus on analysing accounts of self-injury provided in a particular context (an interview with me). The commonalities between the accounts provided, and especially their parallels with other work exploring illness narratives, demonstrate the importance of wider cultural resources in shaping the ways in which self-injury is understood.

Analysing self-injury using the concept of illness narratives may not, at first glance, seem appropriate. Contested as it is, self-injury is not necessarily an *illness*, though some would argue it should be seen as such: ‘non-suicidal self-injury’ has recently been proposed as a psychiatric diagnosis and it remains unclear how such changes in categorisation might shape individual understandings.^1^ ^25^ Further, while illness narratives have been largely used to explore accounts of chronic conditions such as spinal cord injury or cancer,^26^ ^27^ individual acts of self-injury might more accurately be described as acute. Nevertheless, for some, the practice of self-injury can be experienced as compulsive and difficult to stop;^4^ therefore, *repeated* self-injury could perhaps be described as chronic. In some cases, the consequences of self-injury include long-lasting, permanent marks and scars. Thus, even when individuals have effectively ‘stopped’ injuring themselves, they may carry noticeable evidence of their past behaviour; as such, the visible, corporeal effects of self-injury in the form of scarring may also be understood as chronic.

Narrative approaches to the study of self-harm (self-injury *and* self-poisoning) have indicated the importance and diversity of different modes of accounting for the practice. Written accounts of self-injury were examined by Boynton and Auerbach^28^ among teenagers, and Harris^3^ among adult women. These analyses demonstrated the wide range of ways in which narratives of self-injury were constructed and situated within broader cultural framings regarding gender, bodies, spirituality, punishment and pleasure. Accounts of the experience of living with a body marked by self-injury have been little discussed in existing literature. Additionally, while research has clearly highlighted the rich and diverse meanings expressed via narratives about self-injury, it has focused largely on the voices of women or those in clinical treatment.^3^ ^28^ ^29^ This paper builds upon previous work, exploring life-story narratives of living with a self-injuring and self-injured body, among a diverse group of men and women. Leading from the finding of Sinclair and Green^29^ that Frank's typology of illness^30^ narratives provided a useful framework for accounts of moving away from self-harm, I demonstrate that this typology can be extended, with some modification, to illuminate accounts of living with a body that has been self-injured. Frank's^30^ approach is particularly well suited to exploring accounts of self-injury because it invites reflection on embodied experience, and on the intimate relationship between bodies and narrative. My application of Frank's typology of illness narratives (quest, chaos, restitution)^30^ to self-injury partially addresses calls for the use of phenomenological approaches to understand illness experience,^31^ ^32^ demonstrating the salience of this method for those whose bodies are permanently marked by a practice viewed by many as pathological.

## Listening to narratives of self-injury

The narratives discussed here were generated during research that aimed to explore the ‘lived experience’ of self-injury, using life-story interviews with 12 people who had self-injured. Participants were recruited through community sites in Scotland, UK, and related diverse experiences with self-injury and with formal support services. Between 2007 and 2008, each person was interviewed twice, with the first interview focusing on their ‘life story’, and the second exploring their understanding and experiences of self-injury more explicitly. Interviewees were aged between 21 and 37; five were men and seven women. Of the 12 participants, eight suggested that they had ‘stopped’ injuring themselves, between 1 and 8 years prior to the research. Four indicated that they continued to injure themselves, and all four reported doing so between the two interviews. Interviews were recorded and transcribed verbatim. Analysis incorporated thematic and narrative approaches, informed by sociological theorisation on emotion and embodiment.^22^ ^33^

The research was approved by the University of Edinburgh ethics committee (School of Social and Political Science). All participants provided informed, written consent including consent to reproduce quotations from the interviews in published work. Participants were given the opportunity to read transcripts, though only one participant took this up. The second interview provided further opportunity to encourage active engagement in the narratives being produced during the research; in the second interview, participants were invited to contribute their own themes for discussion. This reflected the original aims of the project which had been collaborative,^34^ though in practice this did not work out as planned (see 35).

The analysis presented here is based on naturalised transcriptions of interview discussions.^36^ Thus, the analysis might be said to focus on what Frank called ‘enacted’ stories (p. 116),^30^ though these stories were generated artfully in a research interview. During data collection, transcription and analysis, I was concerned with *how* self-injury was talked about and in order to do this, it seemed important to preserve, as far as possible, the manner in which participants told their stories. These accounts are different, then, from many of the published illness narratives Frank drew on when he set out a typology of illness narratives in *The Wounded Storyteller*.^30^ The accounts I discuss here are certainly ‘messier’; they represent stories told at a specific point in time, to a particular person. They may not be the stories that participants would tell now.

Despite the ‘messiness’ of participants’ accounts, early on in analysis I began to identify commonalities and contrasts in how talk about self-injury, and self-injury scars, was structured. Particularly with regard to accounts of self-injured bodies, Frank's typology of illness narratives (chaos, quest and restitution) provides a useful approach to exploring these structures.^30^ As with other studies using this typology,^27^ ^37^ the boundaries between the three types were not always clear and participants’ accounts often contained elements of all three. The most frequently provided narrative incorporated both quest and restitution narratives. Typically, this entailed participants emphasising their lack of regret over their past practice of self-injury, suggesting involvement in the practice had ultimately changed either the individual or a situation for the better. However, alongside this, participants highlighted ambivalent feelings about scars, and detailed attempts they had made to remove, minimise or obscure scars. In common with previous research on illness narratives,^26^ chaos narratives were less common, with only one participant's narrative aligning closely with this type.

## Restitution: returning to a preself-injured state

Restitution narratives address a desire for a return to a preillness, or preinjury, state. While in some cases (eg, spinal cord injury^26^) such a return may be extremely unlikely, the wish and hope to do so nevertheless form an important aspect of the overall narrative. Six participants alluded to ideas of returning the body to a preself-injured state by either concealing scarring with tattoos or undergoing surgical interventions to minimise them. However, in most of these accounts scars were discussed with some ambivalence, with participants’ accounts indicating little commitment to removing scars entirely. Only one participant, Justin, provided a dominant restitution narrative. In most other cases, participants suggested that they did not ‘mind’ their scars, but simultaneously indicated concern and anxiety around what others might think—or assume—on seeing scars.

I suppose there's a bit of disparity cos, in my mind I sort of feel like I'm OK with it, like, I'm perfectly, happy with, […] what I've, you know I don't have, any reg- I don't really regret doing it or I'm, really ashamed of it or, you know anything like that, but at the same time I'm not … I don't, wouldn't want to just openly talk about it at work […] I think that's basically cos of, I think they might have preconceptions. *Francis*

Francis did not talk explicitly about removing his scars, though he did describe being cautious about when they were revealed. Careful management of the visibility of self-injury scars was common across the sample, and appeared to lead from concerns about the perceptions of others. Such concerns also seemed to underlie accounts that explicitly addressed scar removal. Justin's narrative provided a detailed and involved account of his efforts to remove and conceal scarring to both of his arms:

I also looked into like you know, trying to see, er, ways of kind of you know, making scar, tissue look less, obvious and stuff erm, … I got this quite interesting stuff that was like em, … kind of like em, a gel pad, a silicone gel pad […] that kind of, comp[ressed] and actually, made- you know you had to wear it, like every night […] and then, like it consistently kind of pushed it down […] but then if you don't keep using it you know it sort of, they sort of show more […] and you end up kinda going back to the, state […] but, em, that flattened it off […]so that, you know that was again, kind of, you know trying to kind of, get to the point where you don't feel kind of worried about kind of…. *Justin*

Justin described going on to get a large tattoo over the now flattened scars in order to further conceal the marks. This was the most unequivocal account of removing scarring caused by self-injury provided in this study. One other participant, Harriet, described having a medical procedure carried out in order to minimise scarring to her arms. Harriet did not detail exactly why she had undergone the procedure, but elsewhere in her account she suggested a commitment to *continuing* to self-injure, emphasising the importance of hiding this in order to avoid interference from others. While Justin's narrative indicated an overall desire to have his body reflect his current status as someone who did not self-injure, Harriet's indicated a wish to continue self-injuring without undue interference, maintaining an impression that she no longer self-injured while continuing to do so in a more hidden manner. The ‘fix’ being discussed in each of these accounts is not the practice of self-injury, but rather, the enduring aftermath.

With the exception of Justin and perhaps Harriet, participants’ accounts of scar removal or minimisation tended to be more ambivalent. These narratives referred to attempts to minimise or conceal scars, while simultaneously affirming that they sometimes felt confident or comfortable with them.

[a friend] once asked me, if, … if I could, go back again, … you know, if I was actually embarrassed by, … my scars and things and, …and if, it, … Em [pause] you know if I would do it again if I went back […] and I said, I probably would, still do it but, ... I do kind of regret having done it, at the same time, em [pause] but [pause] it was a part of my life for, [pause] a good, …10 years, so, … em, [pause] well, a very bad 10 years actually not a very good 10 years [*later*] I do regret the fact that I have so many scars that I can't [pause] you know, that I can't wear t-shirts around my parents. *Emma*

As Emma reflected on this remembered exchange she was hesitant, noting that while she would not want to change anything about her past practice of self-injury, she nevertheless regretted the visible marks it had left, which she felt she had to continue to conceal from her parents. Other participants talked similarly about carefully choosing when and where to reveal or hide their scars.

Restitution narratives are portrayed as representing a medicalised approach to illness—one that searches for a cure or ‘fix’ for the illness or problem.^26–28^ The restitution narrative is understood to cohere closely with modernist expectations that illnesses can be cured or fixed.^37^ With self-injury, where there is permanent scarring, such a fix may be practically impossible. Given the difficulty of entirely removing or concealing scars, it may be that people who carry such marks are therefore more inclined to provide accounts which defend their existence. Indeed, this was at least a possibility for most, as scars left by self-injury were not described as inherently problematic. Unlike the illnesses, injuries and conditions addressed in other studies using the typology,^27^ ^30^ ^37^ scars themselves did not cause discomfort. Nonetheless, they *were* framed as problematic, requiring management, attention and accounting for.

In this study, although all participants talked about concealing scars—occasionally permanently—only Justin appeared to have made a concerted effort to remove all trace of them. Others, as with Emma and Francis, were far more ambivalent, and while they might conceal them in certain situations, removing their scars outright was not a feature of their narrative. This underlines the potential importance of the presence of long-term scarring in shaping the possible narratives available to those who have self-injured, and perhaps suggests that such scars position self-injury alongside other chronic conditions which similarly struggle to maintain a restitution narrative.^37^ Importantly, participants did not provide restitution narratives about ongoing self-injury and, as demonstrated though Harriet's account, it was possible to provide an account of medical intervention to remove scarring, while actively self-injuring.

## Chaotic bodies: gaining and losing control

There is a difference between the ambivalence expressed by Francis and Emma, and the more explicitly negative—perhaps chaotic—account provided by Anna. The chaos narrative is one of the more challenging of Frank's typology.^30^ Frank argued that narratives characterised by chaos indicate a *lack* of narrative, an absence of coherence to the events or experiences being related: ‘lived chaos makes reflection, and consequently story-telling, impossible’ (p. 98).^30^ Chaos in illness narratives infers a lack of hope, and a lack of control over the events befalling the teller. As with the study by Sparkes and Smith^26^ of narratives of spinal cord injury, only one participant provided a narrative that adhered to a more chaotic form when discussing living with a self-injured body. Chaos, in Anna's narrative, was reflected in her orientation towards the future, as well as her description of her body, and the scars it carried. Other participants’ accounts were often typified by chaos when talking about their early experiences with self-injury. In each case, self-injury was described as a response to chaos, a way of coping with a chaotic situation. Only in Anna's account did the chaos appear to extend to the aftermath of self-injury as well.

Anna, like some of those described above, indicated some attempt to remove the scars generated by her practice of self-injury. However, in contrast, she emphasised the futility of her efforts. More importantly, she reflected that the presence of her scars provided a reason to *continue* to self-injure:

… the scars are there for, forever now, so [pause] I think that's kinda a bad thing though, because it, … see if it's something that faded over time, you might sorta go, oh well, it all faded so, that's it I'll no bother. But I've got these scars now, they're there now, the damage is done, I just cut on top eh scars now, just, covered… totally utterly covered [pause] so it's like, phew [pause] what's the point, of stopping. *Anna*

Anna suggests therefore that the nature and extent of her scars provided a reason not to stop—‘what's the point’. Anna's discussion of her scars reflected her broader narrative which was often pessimistic in relation to her life in general, reflected also in her accounts about her body. She described herself as having an intensely uncomfortable relationship with her body, which was manifested in feelings of self-loathing and disgust, and practices which, as well as cutting herself, included disordered eating.

I just have this, sortae warped body image, and I don't know if that's, again, I don't know if that's part ae the ... the self-harm, d'you know, I don't know if that's why [pause] like I hate this body so I'll just, [pause] abuse it [laughs] […] I mean I cannae, can't look in the mirror, cannae look at myself [long pause] just, disgusting. *Anna*

Anna's account here and during the previous excerpt was uncertain and hesitant; her tone markedly deflated. These more negative sections of Anna's interviews aligned closely with the chaos narrative form, lacking focus and hope, providing a sense that the teller did not feel ‘in control’ of the situations she described. Anna did not present a narrative which was wholly ‘in chaos’, however, and she provided a more hopeful account at other times in her interviews. In particular, at some points her narrative indicated her practice of self-injury might provide an *escape* from chaos. While Anna suggested her self-injury related to self-hatred, elsewhere in her interviews, self-injury was framed as an act carried out in response to overwhelming emotional and social situations, where she felt out of control. Self-injury, at times, offered a way to regain control and—perhaps—to conquer chaos, if only temporarily.

If I'm no’ in control of a situation, or if I'm no’ in control of what's happenin’ … that's when I self-harm […] It's like… if, if somebody says something or, or [pause] or… you know something's going on and I'm like ‘oh god I cannae stop this’ or … em sometimes I start to panic aboot things, and the only way I can stop panicking about it and think rationally about it is … cut myself [pause] it's just like, I dunno it makes me just stop I suppose and then, it's like right ok, deal wi it. So I think it's like getting control or gaining control. *Anna*

Self-injury was described similarly by a number of other participants, and ‘control’ was certainly a recurring motif throughout the interviews when describing the *practice* of self-injury. Control is also an important feature of Frank's illness narratives, both in terms of implied control (or lack of control) of the body and as regards the use of story and narrative as a way of regaining control over the ill body.^30^ In Anna's narrative, self-injury is a response to chaos, but also contributes to ongoing chaos: generating further scars, further wounds. While Anna described self-injury as a way of gaining control, and emphasised her need to feel ‘in control’, she also alluded to a lack of control, both regarding the act of self-injury and the corporeal aftermath.

Have you seen that [scar reduction product] that's advertised? […] it kinda does fade them, but, ‘fraid I think I've got too many big, deep, ... kinda big scars now that it just, it wouldnae work. Em, but for a long time I could get away with [shorter] sleeves cos it wasnae, kinda here, but, it—progresses. *Anna*

Anna's account implied less control over the *progression* of self-injury, and the generation of ‘bigger’, ‘deeper’ scars: scars which were less amenable to attempts to reduce their appearance. Thus, as with the restitution narratives discussed above, Anna's chaos narrative applied particularly to her account of her scars, with chaos being more complicated when describing the act of self-injury itself.

## Transforming the self: re-visioning scars

In stark contrast to Anna's account, several participants provided narratives about their practice of self-injury *and* their permanent scarring, which emphasised the transformative, positive nature of both. These narratives align closely with Frank's quest narrative form, as the illness experience is reworked by the teller as initiating a transformed, improved self.^30^ Two participant's narratives indicated that the transformative quality of self-injury originated in the act itself, and their stories tied current, positive, interpretations of scars to the meanings of the initial injury. Another participant spoke of the importance of revealing her scars to others, as a form of reaching out and reassuring others.

Mark provided a narrative which frequently alluded to how self-injury had been effective at the time, helping him to manage periods of depression: ‘it worked, it worked […] it's always had a positive, feeling to me’. This affirmative account was carried through into Mark's discussion of the scars that his practice of self-injury had left:

But, because that one was so bad, em, … it almost serves as, as a [sign] I don't need to cut, I've got that […] it's like er, it's like a badge. […] I think if I hadn't done that, my arm would have been a lot more—covered in small cuts. *Mark*

This particular narrative referred to a large scar left by what Mark indicated had been his final act of self-injury. Mark portrayed this event (cutting himself, ‘badly’) as effectively ending a difficult interpersonal relationship. As indicated here, he suggested that the resulting scar now acted as a signal, or reminder, that he did not ‘need to cut’. Significantly, Mark's account argues that had he not cut himself ‘badly’ on that occasion, his body may have now carried numerous smaller scars. Mark's discussion paralleled those provided by others where scars, and the self-injury which had generated them, were linguistically harnessed in order to generate an understandable, meaningful, account of both past acts and current, scarred, body.

In Rease's case, self-injury was explicitly framed as an important part of a broader transformation, helping her to feel more comfortable in her own body, as well as being a response to feelings of anger, self-loathing and depression.

It's [depression] like you're, cut-off from people. So I felt like that, and the, the self-harm brought me back to life[…] would kinda wake me up, and just make me feel so much better. *Rease*

Rease argued that both her earlier practice of self-injury and the scars she carried with her in the present were positive and represented constructive acts, involving taking control of her body, her life and her story:

… it is about adornment and celebration […] And in a way my scars are as well, actually, ‘cos I do think they're really beautiful, and they're like a part of my, my experience, my history. And I very much believe about, em, your experience—written on the body and the body telling a story. *Rease*

While other participants similarly emphasised that self-injury had been a successful method of managing distress, the accounts of Rease and Mark differed in explicitly tying positive meanings to both their practice of self-injury *and* the resulting scars.

That scars and the body might tell a story provoke questions about *who* the story might be for, and whether others might understand the story in the way the teller/body intends. Indeed, the accounts participants provided about hiding, concealing or minimising their scars frequently alluded to concerns about how ‘others’ might read scars. A contrast to this concern is found in Milly's account of deciding to ‘stop hiding’ her scars. Like Rease and Mark, Milly provided a provocative narrative, where she subverted concepts of stigma and shame, suggesting that viewing her scars could act as a form of support for others who might not be ready to be as open as she was:

I, for me it's a sense of pride, of being able to say to people ‘I've, been through crap, but I've got over it’ rather than keeping it hidden […later…] to be able to show, and I don't think this has, been discussed either, to be able to show, what I've done, it's not—like I said earlier on—it's not like ‘hey look at me, look what I've been through […] isn't it shit’ … it's a, … this is, this is what I have [been through], this is what I was, and this is who I am now. *Milly*

Milly framed her revealing of her self-injury scars as a moral, compassionate move that opened up conversations with others who had self-injured and facilitated sharing of experiences. She emphasised her ‘pride’ in who she was, contrasting this with earlier difficulties she had faced during adolescence and young adulthood.

The quest narratives produced during this research provide parallels to Frank's discussion of the ethics of storytelling, and particularly the ethics invoked by quest narratives.^30^ The accounts of Rease, Mark and Milly touched on an *ethics of recollection*, of *solidarity and commitment*, and of *inspiration* (Frank^30^ pp. 132–33): Rease and Mark highlighted the importance of scars in anchoring memories of past actions, while Milly's account emphasised the centrality of scars in developing shared understandings and of inspiring others to live confidently with their own marked body. These narratives might be seen to reflect the communicative body, in action.^30^

### Reading and listening to the self-injured body

The accounts discussed in this paper demonstrate the diverse meanings that self-injury, and the scars that it leaves, can hold. Although self-injury is not straight-forwardly an ‘illness’, accounts of self-injury reflect Frank's typology of illness narratives, particularly when attention is paid to narratives about bodies that have been scarred by self-injury.^30^ It is less clear that illness narratives are an appropriate lens through which to understand the *practice* of self-injury. As such, this analysis parallels the use by Sinclair and Green of the typology to analyse accounts of moving away from self-injury,^29^ though in this paper I focus in particular on accounts of the embodied aspects of being someone who has self-injured, or who still does.

While illness narratives frequently refer to or invoke the ill body, with self-injury the scars—the evidence of ‘illness’—can be both the starting point and originator of the story. Participants described deep unease about the possibility that others might ‘read’ scars incorrectly, or might make unfavourable assumptions about them as a result of seeing them; even those who provided positive accounts of scars indicated that they concealed them in certain contexts. Thus, the scars left by self-injury can be understood themselves as communicative, and narratives provided by people who are scarred provide an opportunity to control, to some extent, the nature of this communication. The analysis developed here indicates that the level of control people might have over these narratives varies, though in all cases drawing on culturally available frameworks: of overcoming and transforming bodies and stories through painful experience; of feeling out-of-control and losing hope; of gaining control via interventions and ‘fixes’ which return the body—at least partially—to what it once was.

Attending to the diverse ways in which scars—and self-injury—may be understood should comprise an important aspect of compassionate clinical practice. Carel^31^ has recently argued for the importance of phenomenological approaches to improving medical practice and research, suggesting that paying attention to embodiment provides a more holistic view of illness experience. While Carel suggests that narrative approaches often fall short of adequately incorporating the body, Frank's typology of illness narratives addresses bodies directly.^30^ The analysis presented here has focused on accounts of living in and with a body scarred by self-injury, thus providing a *partially* embodied perspective on this experience. Further, by highlighting accounts of the impact of living with a self-injured body, our attention is drawn to the importance of the long-term nature of some self-injury in which scars may endure long after the practice itself has ceased. Given the apparent rise in the number of people who are engaging in self-injury,^6^ ^12^ it seems likely that medical practitioners will come across individuals marked by self-injury in greater frequency. A phenomenological, narrative approach demonstrates that care should be taken not to make assumptions about what these marks might mean for individual patients.

The existence of permanent scarring following self-injury invokes different types of account. This paper has explored three of these among a relatively small sample of adults, following Frank's typology of chaos, restitution and quest.^30^ Future work with the narratives of people who have self-injured should explore this further in order to ascertain whether this analysis is more widely applicable, and whether among other samples the typology might be more appropriate for the practice, as well as the aftermath, of self-injury. There are numerous factors which might shape the way in which scars left by self-injury are narrated and accounted for. Certainly, how recently a person has self-injured may help to explain some of this diversity. Chaos narratives, like Anna's, may be more likely if self-injury is an ongoing concern. It is also possible that the nature and setting of the research interview encourages particular forms of narrative. Interviews in this study were organised around a discussion of the participants’ ‘life story’ and there may have been an impulse in providing such an account to give a positive ending. Indeed, this may well be the case with much interview-based research, as noted by Bury.^24^ This raises questions as to the extent to which qualitative interview studies provide adequate space or opportunity for more pessimistic, chaotic stories.
